# Multi-band MIM refractive index biosensor based on Ag-air grating with equivalent circuit and T-matrix methods in near-infrared region

**DOI:** 10.1038/s41598-020-63459-w

**Published:** 2020-04-14

**Authors:** Mohamad Nejat, Najmeh Nozhat

**Affiliations:** Department of Electrical Engineering, Shiraz University of Technology, Shiraz, 7155713876 Iran

**Keywords:** Nanophotonics and plasmonics, Optical sensors

## Abstract

In this paper, a multi-band metal-insulator-metal (MIM) perfect absorber with refractive index sensing capability has been investigated in near-infrared region. The proposed structure has been studied for biomedical applications such as detection of solution of glucose in water, diagnosis of different stages of malaria infection, bacillus bacteria and cancer cells. The MIM configuration improves the sensing parameters of the biosensor due to the good interaction with the analyte. The high sensitivity and figure of merit of 2000 nm/RIU and 100 RIU^−1^ have been achieved, respectively. Also, the Ag-air grating in the suggested plasmonic sensor helps the localized surface plasmons excitation and makes the structure sensitive to the incident lightwave polarization. Therefore, the presented biosensor behaves like a polarization switch with the high extinction ratio and fast response time of 25.15 dB and 100 fs, respectively. The methods of equivalent circuit model and transmission matrix have been utilized to verify the simulation results, as a new challenge in near-infrared region. The new idea of multi-application plasmonic devices, the feasibility of fabrication for the presented structure and utilizing mentioned analytical methods in near-infrared region could pave the way for the future of plasmonic structures.

## Introduction

Surface plasmons (SPs), which are created by the interaction between the free electrons of metal and incident electromagnetic wave at metal-dielectric interfaces, have two kinds of localized surface plasmons (LSPs) and propagating surface plasmons (PSPs)^1^. While LSPs only oscillate on their own place, PSPs propagate in the form of an oscillating charge wave due to the large enough interface of the metallic layer^2^. Today, the theory of surface plasmon resonance (SPR) has been used in a lot of optical studies about food safety testing^3^, environmental monitoring^4^, medical diagnosis^5^, optical bistability^6^, surface-enhanced Raman spectroscopy (SERS)^7^, and second harmonic (SH) generation^8^. Subwavelength structures with metal films, which usually form a nanocavity, allow the hybridization of the cavity resonance modes with the LSP modes^2^. Overcoming the diffraction limit and field enhancement are other benefits of plasmonic structures^9^.

Over recent years, many SP-based structures have been studied and investigated such as perfect absorbers^10^, waveguides^11^, modulators^12^, lasers^13^, and sensors^14^. The unique properties of the SPRs such as strong dependency on the size, geometrical shape and refractive index of the surrounding medium make them good candidates for sensing applications^15^.

It is well known that the sensing performance of SPR-based sensors is due to the wavelength shift $(\Delta \lambda )$ of the response spectrum, when the refractive index of the test medium $(\Delta n)$ changes. The sensitivity $({\rm{S}}=\Delta \lambda /\Delta n)$, full-width at half-maximum (FWHM) and figure of merit (FOM = S/FWHM) are the main parameters for evaluating the sensing performance^14,16^. SPR-based sensors have attracted much attention because of good advantages like miniaturization, rapid response and high sensitivity^17^. Moreover, they have a key role in detection of bacteria^18^, proteins^19^, DNA^20^, RNA^21^, viruses^22^, analytes^23^, and chemical and biological species^24^.

Widespread applications such as food quality control, health, diseases diagnosis, and environmental and molecular monitoring make the biosensors a hot topic for researchers in these years^25–27^. The label-based and label-free sensors are two main types of biosensors. The first one has lower reliability than the second one because it can change the molecule’s binding properties. The label-free biosensors, which have advantages such as real time monitoring without any label, operate like other SPR-based sensors and detect the refractive index variation of the test medium^28^. Label-free detection, direct and rapid response, spectral tunability, strong enhancement of local electric field, and adaptability to modern nanotechnology architectures are some of the remarkable benefits of the SPR-based label-free biosensors that have been widely studied for medical applications^28,29^.

Sun *et al*. have suggested a plasmonic sensor based on Mach-Zehnder interferometer with a double-slot hybrid plasmonic waveguide and the high sensitivity of 1061 nm/RIU has been attained^30^. Also, a horizontal slot waveguide biosensor for detection of DNA hybridization with the sensitivity of 893.5 nm/RIU has been investigated^31^. Another label-free plasmonic biosensor has been studied by Hameed *et al*.^28^. Their suggested structure consists of a hybrid plasmonic slot waveguide based on silicon-on-insulator (SOI) and the high sensitivity of 1890.4 nm/RIU has been achieved for detection of DNA hybridization. Another group of plasmonic sensors are metamaterial based ones. Kabashin *et al*. have presented a metamaterial based plasmonic sensor for biosensing applications^32^. They have illustrated an improvement in biosensing technology using a plasmonic metamaterial, which supports a guided mode in a porous nanorod layer. Moreover, a microfluidic sensor for dielectric characterization has been studied and presented in ref. ^33^. The proposed sensor consists of a split ring resonator, which can help the use of microfluidic sensors for identification, classification, and characterization of chemical and biochemical analytes.

There are different methods to excite SPs in plasmonic structures. The nanoparticle usage for LSPs excitation is the primary method^1^. It is worth noting that according to the definition of LSPs, an arbitrary dielectric-metal interface with smaller length than the resonance wavelength can provide a context in which the excitation of LSPs occurs. Prism-coupling, waveguide-coupling, and grating-coupling are some well-known methods for PSPs excitation. A conventional plasmonic structure for PSPs excitation is Kretschmann configuration, which has a thin metal layer coated over the base of a prism^34^. For a long time, the Kretschmann structure has been studied and commercialized, but the need for the prism makes it bulky. Therefore, a compact and portable sensor with the ability of integration with other plasmonic devices is needed. The grating-based plasmonic structure that has been studied in many researches can satisfy the mentioned requirements^9,35^.

Metal-insulator-metal (MIM) configuration which has been utilized former in many structures such as waveguides, filters, switches and interferometers can enhance the sensing performance of plasmonic sensors^17^. For example, Xie *et al*. have suggested a plasmonic sensor based on MIM waveguide with side-coupled hexagonal cavity^36^. They have achieved the high sensitivity of 1562.5 nm/RIU and good FOM of 38.6 RIU^−1^ at *λ* = 1550 nm.

In this paper, a hybrid plasmonic four-band perfect absorber has been investigated as a biosensor for detection of malaria infection, cancer cells, bacillus bacteria, and 25% solution of glucose in water in near-infrared region. First, the design procedure of the multi-band absorber with sensing capability has been studied based on the MIM configuration and Ag-air grating. Then, the effects of geometrical parameters on the absorption and sensing performances, the physical insight of perfect absorption and the fabrication process have been investigated. Also, two analytical methods of equivalent circuit model and transmission (*ABCD*) matrix have been utilized to verify the simulation results. Moreover, the switching performance of the proposed structure has been shown. Here, our new idea of multi-application plasmonic devices has been utilized to have sensing and switching performances in the designed structure, simultaneously. The achievements can pave the way for utilizing two mentioned analytical methods to validate the simulation results of any arbitrary plasmonic structure at near-infrared wavelengths. In addition, the good performances of our proposed structure in sensing and switching capabilities can open a new window for development of plasmonic applications.

## Design and Simulation Results of Four-band Perfect Absorber with Sensing Performance

**Figure 1:**
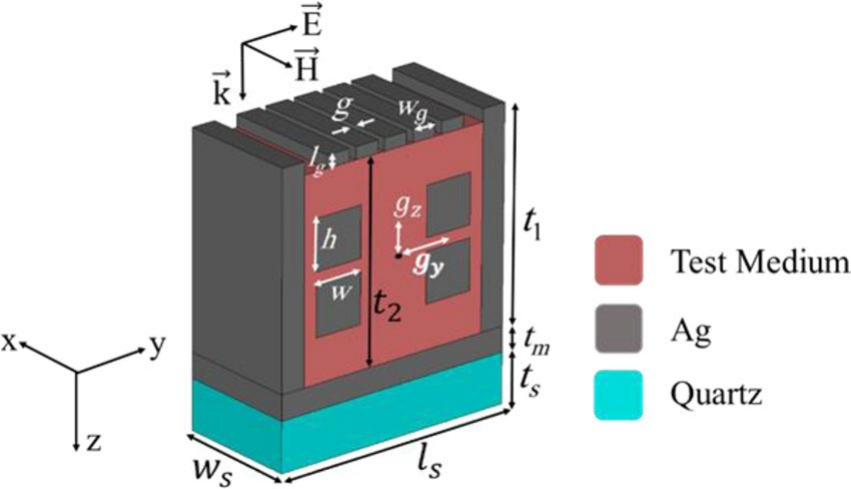
The schematic view of a unit cell of the proposed biosensor.

A unit cell of the proposed structure that is composed of a dielectric substrate, a layer of silver (Ag) and silver walls is depicted in Fig. 1. The Ag walls help the perfect absorption of the structure by trapping the incident lightwave and causing more SPRs. More importantly, these walls provide a context in which the fluid test substance can cross in the designated path. To have good interaction with the analyte, four Ag cubic resonators are located inside the suggested sensor, where is filled with the test material. The arrangement of the Ag cubic resonators and other parts of the structure causes the MIM coupling effect in the presented structure. Also, an Ag-air grating with the period of *w*_*g*_ + *g* = 130 nm is used on top of the structure to help the SPs excitation, when the incident lightwave impinges on the structure.

The dielectric layer is quartz with the permittivity of *ε*_*r*_ = 2.25 and the silver is modeled according to the Johnson and Christy data^37,38^. The absorption coefficient (*A*) can be given by^39^:

1 $$A=1-R-T$$

where *T* and *R* are the transmission and reflection coefficients, respectively.

The CST Microwave Studio software has been used to perform the full-wave simulation based on the three dimensional (3D) finite element method (FEM) with open boundary condition along the z direction and periodic boundary condition along the x and y directions. The open boundary condition is one of the most useful boundary conditions in this software which has been used along the direction of the incident lightwave (z-axis) to provide the stimulation conditions and consequently the source can be applied in this direction. The tetrahedral mesh in the frequency domain with the size of 10 tetrahedrons per wavelength has been utilized to calculate the scattering parameters and absorption spectrum according to Eq. (1). All the remained parameters in the simulation are selected as the default setting of the software.

When an electromagnetic wave illuminates the structure from the top, it will be trapped in the absorber, since the thickness of the Ag layer is larger than its skin depth in near-infrared region (*T* = 0). The y-polarized incident wave excites the SPs by means of the Ag-air grating. The SPs excitation and the MIM coupling help the perfect absorption by absorbing the energy of the incident lightwave. Consequently, by selecting the values of the geometrical parameters according to Table 1, the structure can behave as a multi-band perfect absorber, as it will be shown.

**Table 1:** The values of the geometrical parameters of the proposed four-band biosensor.

Parameter	Value (nm)	Parameter	Value (nm)
*w*_*s*_	500	*g*_*y*_	250
*l*_*s*_	1010	*g*_*z*_	125
*t*_*s*_	200	*h*	200
*t*_*m*_	100	*w*	200
*t*_1_	850	*l*_*g*_	50
*t*_2_	800	*w*_*g*_	100

**Figure 2:**
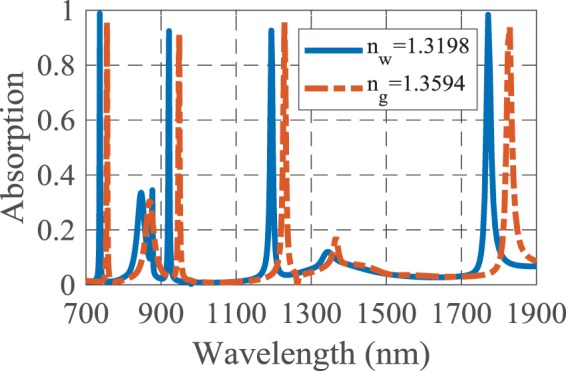
The absorption spectra of the suggested biosensor when the analyte refractive index changes for detection of 25% solution of glucose in water.

According to the dependency of SPRs properties on the surrounding medium, a change in the refractive index of the analyte results in a change in the absorption spectrum of the structure. Here, the absorption spectrum experiences a wavelength shift by changing the refractive index of the test medium from *n*_*w*_ = 1.3198 to *n*_*g*_ = 1.3594, as shown in Fig. 2.

The proposed structure has four near-unity absorption peaks at the wavelengths of 745, 935, 1207.5 and 1800 nm. The sensitivity and FOM of the presented biosensor are 479.79, 686.86, 878.78 and 1457.57 nm/RIU and 239.89, 171.71, 109.84 and 85.71 RIU^−1^, respectively, for the four mentioned wavelengths. It is noteworthy that *n*_*w*_ and *n*_*g*_ are the refractive indices of water and 25% solution of glucose in water, respectively^2^. Here, the four near perfect absorption are due to the MIM configuration and SPs excitation by utilizing the cubic resonators and Ag-air grating, and also proper values for geometrical parameters of the structure. The physical insight of the light absorption will be discussed explicitly in the following.

**Figure 3:**
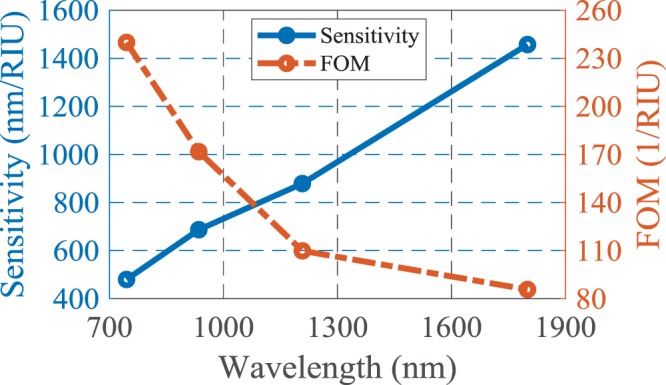
The sensing parameters of the proposed structure at resonance wavelengths.

The variation of the sensitivity and FOM at the resonance wavelengths is illustrated in Fig. 3. It should be noticed that the resonance modes of the structure are decreased by increasing the operating wavelength as a result of the cavity behavior of the structure. Therefore, the sensitivity is improved for higher wavelengths, in which the structure has few resonances and all the absorbed electromagnetic energy contributes to the stronger resonance. However, the loss of the structure is increased and hence the FOM is decreased. In contrast, at lower wavelengths the structure has more resonance modes and so the incident wave energy is divided between more resonance modes and the strength of resonance and sensitivity are decreased. It is obvious that the energy of the incident lightwave for each resonance mode has a particular value.

**Figure 4:**
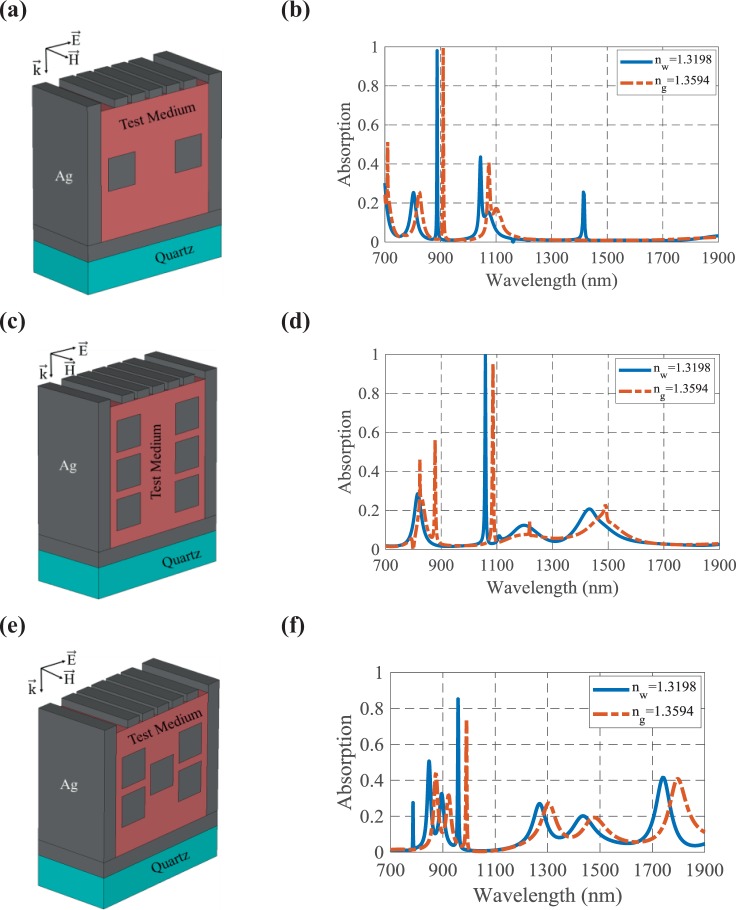
The proposed structure with (**a**) two cubic resonators, (**c**) six cubic resonators, and (**e**) five cubic resonators. The absorption spectra of the biosensor with (**b**) two cubic resonators, (**d**) six cubic resonators, and (**f**) five cubic resonators, when the refractive index changes from *n*_*w*_ = 1.3198 to *n*_*g*_ = 1.3594.

To have a better insight of the cubic resonators effect, we have considered the same structure of Fig. 1 with two resonators while the structural parameters are the same as Table 1. The proposed structure of Fig. 4(a) does not have good resonances due to the large distance between the resonators and other metallic parts of the structure. In this case, the structure has only one perfect absorption peak at *λ* = 900 nm with the sensitivity of 546.71 nm/RIU, as shown in Fig. 4(b). In the second case, the structure of Fig. 1 with six cubic resonators has been considered, as depicted in Fig. 4(c). Since the resonators are so close to each other, the LSPs interaction with the analyte and the sensitivity are enhanced compared to the former state. But, the analyte portion is decreased and the sensitivity is not as good as Fig. 1. According to the absorption spectrum of Fig. 4(d) the sensitivity is obtained as 689.39 nm/RIU at *λ* = 1100 nm. Many other arrangements like Fig. 4(e) have been also simulated and studied. But, as shown in Fig. 4(f), there is no enhancement in the absorbing and sensing performances and the multi-band behavior of the structure is diminished. In this state, the best sensitivity and FOM are 1401.51 nm/RIU and 202.02 RIU^−1^, respectively. The achieved sensing parameters in this state are lower than the reported values for Fig. 1. Therefore, the geometrically optimized structure of Fig. 1 has been chosen in the following. Moreover, the space between the resonators has been tuned by changing the parameters of *g*_*y*_ and *g*_*z*_ and the optimized values have been selected and reported in Table 1.

**Figure 5:**
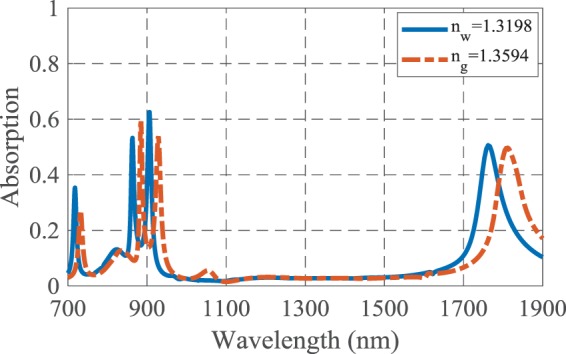
The absorption spectrum of the proposed biosensor without the Ag-air grating for detection of 25% solution of glucose in water.

Here, the effect of Ag-air grating has been investigated. As mentioned before, the grating helps the SPs excitation and the absorption diminishes without the grating, as shown in Fig. 5. It is considerable that the resonances in this case are partly because of the four cubic resonators.

**Figure 6:**
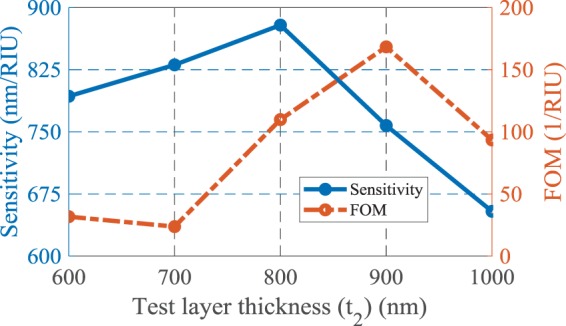
The effect of the test layer thickness on the sensing parameters of the proposed biosensor.

To better understand the effect of MIM configuration and coupling of metallic parts, the test medium thickness (*t*_2_) has been tuned when the other parameters are remained constant. Increasing *t*_2_ results in an increase in the portion of analyte in the structure. Since the proposed structure behaves like a cavity, increasing *t*_2_ leads to improving the sensing performance by providing more space for more resonances and so more interaction between the test medium and excited SPs in the structure. But, as shown in Fig. 6, further increase of the test medium thickness decreases the sensitivity and FOM, due to the less MIM coupling as a result of further gap between the metal parts of the structure. To have good sensing performance *t*_2_ is selected as 800 nm for the following simulations.

**Figure 7:**
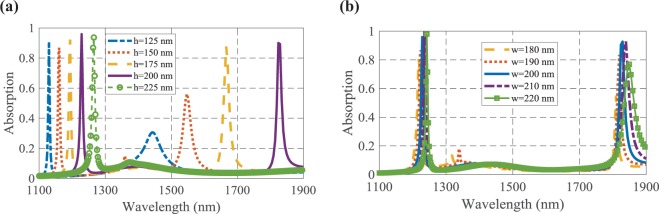
The effects of the (**a**) length and (**b**) width of the resonators on the absorbing function of the proposed biosensor, when the analyte refractive index is 1.3594.

Moreover, the effects of the length (*h*) and width (*w*) of the cubic resonators on the absorbing performance have been studied. According to Fig. 7, it is obvious that increasing the dimensions of the resonators causes a redshift in the resonance wavelength. Since *h* is orthogonal to the electric field of the incident lightwave and the LSPs are almost excited in this interface of metallic cubic resonators and test medium^14^, changing the value of *h* has more effect on the absorption spectrum compared to the variation of *w*. The dimensions of cubic resonators have been chosen 200×200 nm^2^ to create good absorption with proper resonance wavelength.

**Figure 8:**
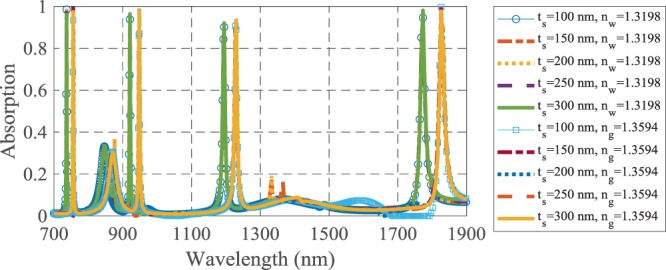
The absorption spectra of the suggested biosensor for different values of the substrate thickness.

The dielectric substrate has been utilized for fabrication feasibility and it does not affect the absorption spectrum, when the Ag layer thickness is more than its skin depth at the desired wavelengths. Therefore, *t*_*s*_ does not have any effect on the absorbing and sensing performances, as shown in Fig. 8.

**Figure 9:**
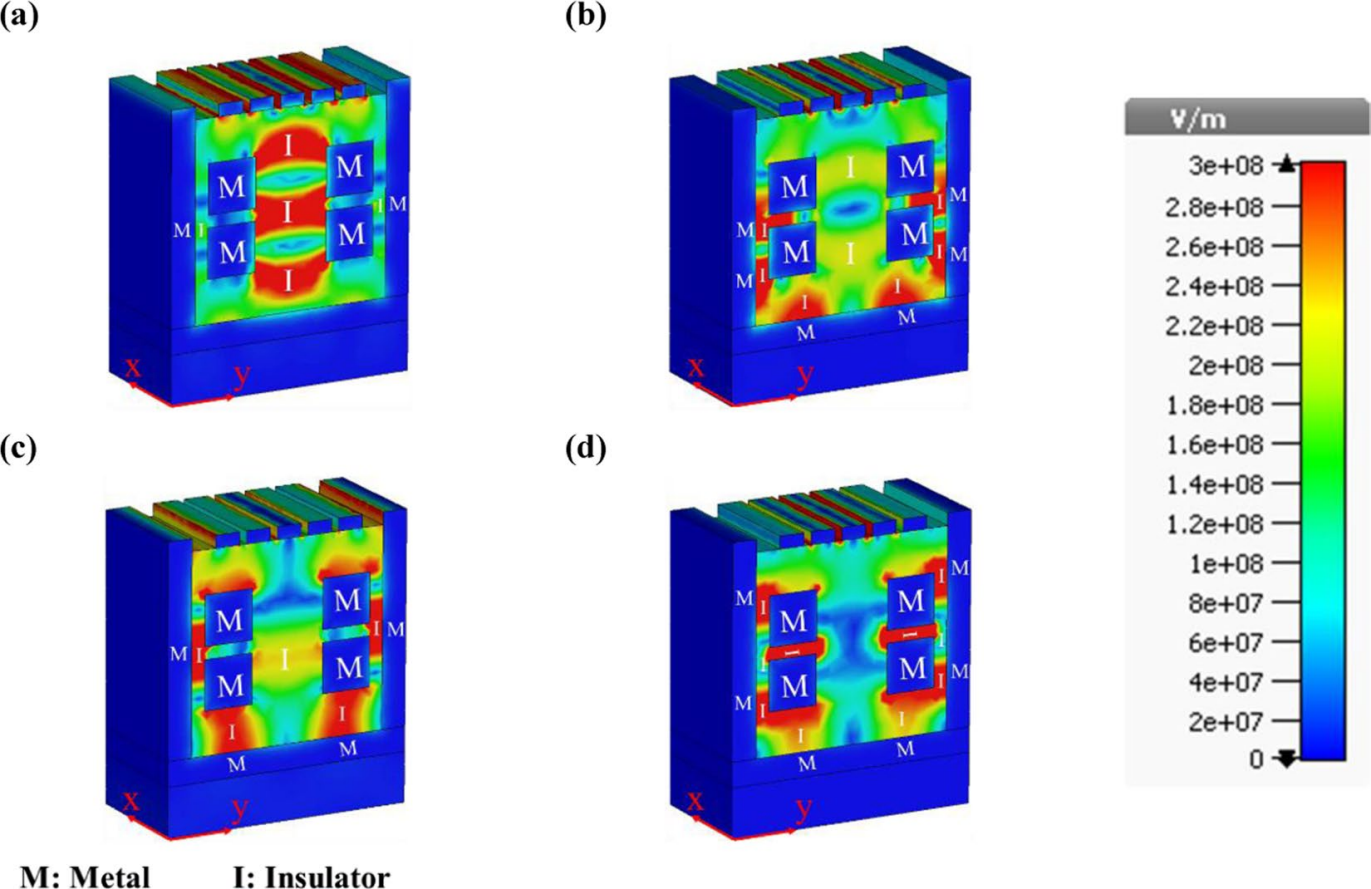
The E-field distributions of the suggested plasmonic sensor at the wavelengths of (**a**) *λ* = 745 nm, (**b**) *λ* = 935 nm, (**c**) *λ* = 1207.5 nm, and (**d**) *λ* = 1800 nm.

Figure 9 demonstrates the E-field distribution $(|\overrightarrow{E}|)$ of the proposed structure for four wavelengths of 745, 935, 1207.5 and 1800 nm. The excitation and interaction of LSPs, which are due to the MIM configuration and Ag-air grating, can be seen in this figure. The MIM coupling of the structure, which has a key role in the performance of the biosensor, causes a good interaction with the test medium as an insulator in the MIM configuration. The cubic shape has been selected for resonators because of the concentration of surface charges at the corners to make the coupling stronger. To have better insight, the MIM parts of the presented structure have been written on Fig. 9. The cubic resonators and the Ag walls and layer are the metal parts, and the test medium is considered as the dielectric part of the structure. Furthermore, all the above mentioned help the perfect absorption performance at four resonance wavelengths. It is obvious from Fig. 9 that the incident lightwave traps in the structure by means of SPRs and good interaction of LSPs which are shown as concentrated E-field distribution at the desired wavelengths.

**Figure 10:**
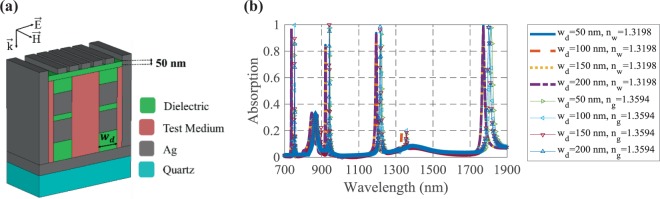
(**a**) The schematic view and (**b**) the absorption spectra of the proposed structure with added dielectric parts.

The fabrication feasibility of the proposed structure has been considered in our design process and consequently some dielectric parts have been added to keep the cubic resonators and make the structure feasible for fabrication. Also, a 50 nm dielectric layer with the refractive index of *n* = 1.4 which can be replaced by LiF in this wavelength range^40^, has been placed under the grating layer to prevent the suspension of Ag-air grating, as shown in Fig. 10(a). The effect of the width of dielectric parts on the absorption spectrum has been also studied in Fig. 10(b) and observed that the added dielectric parts have no significant effect on the performance of the proposed structure.

**Figure 11:**
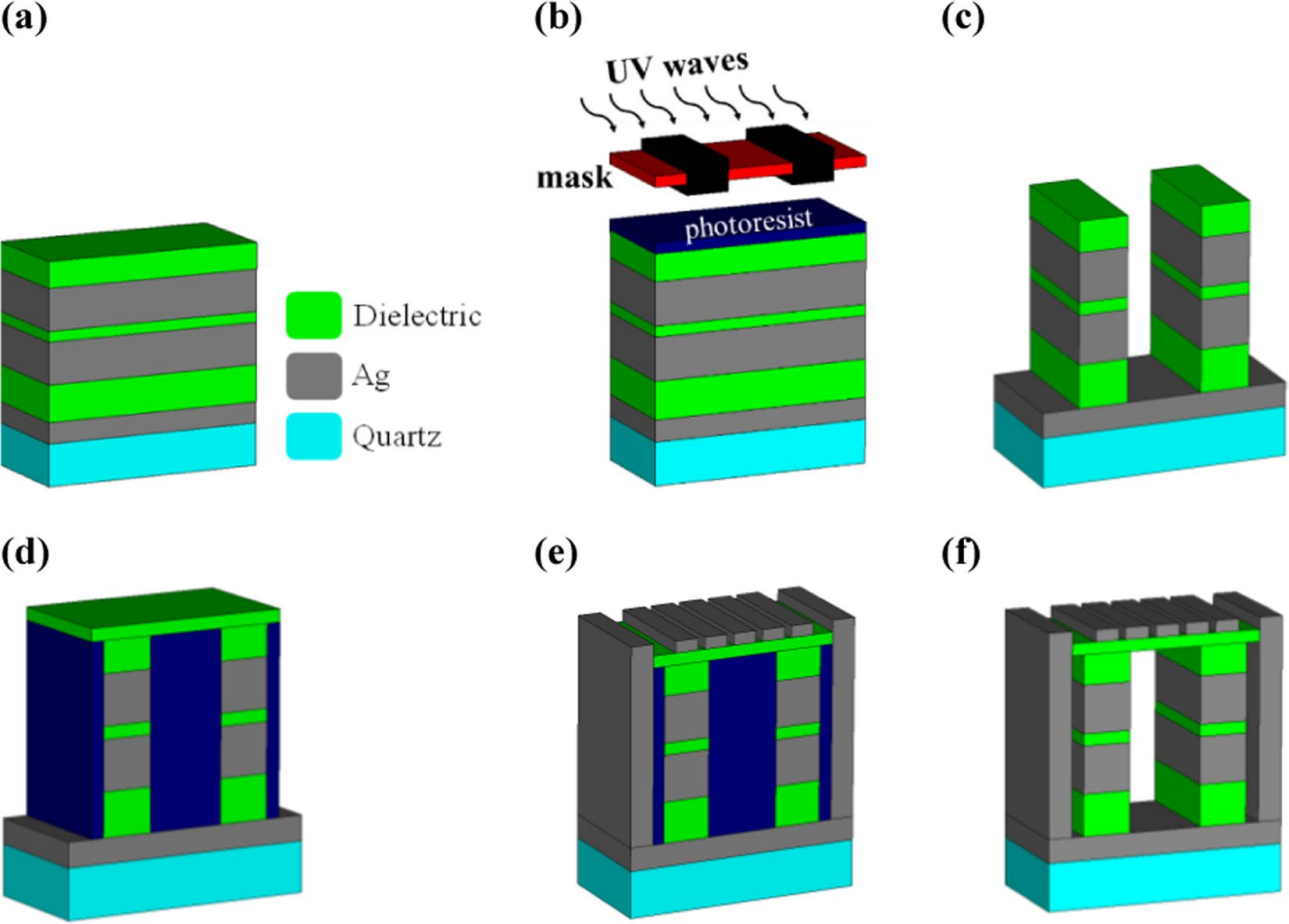
Schematic view of the fabrication process of the suggested plasmonic sensor.

The fabrication process of the proposed structure of Fig. 10(a) can be described as follows. At first, an Ag layer with the thickness of *t*_*m*_ is evaporated on a 200 nm quartz layer. Then, the process goes on with depositing other layers from bottom to top, as shown in Fig. 11(a). According to Fig. 11(b), by means of a mask, deep UV stepper and reactive ion etching (RIE), when a photoresist is placed above the structure, the proposed shape of Fig. 11(c) can be obtained through the lithography method. Afterwards, the test medium of Fig. 10(a) is filled with photoresist as a sacrificial layer and a 50 nm dielectric layer is deposited above the structure, as shown in Fig. 11(d). Next, as depicted in Fig. 11(e), the desired grating and Ag walls are grown and patterned on the top and sides of the structure utilizing lithography and etching. Finally, the sacrificial layer can be removed through the acetone to have the structure of Fig. 11(f).

## The Biomedical Applications of the Proposed Plasmonic Sensor

In this section, the sensing parameters of the proposed plasmonic refractive index sensor in biomedical applications have been investigated. As it was mentioned before, the 25% solution of glucose in water changes the refractive index of water from *n*_*w*_ = 1.3198 to *n*_*g*_ = 1.3594^2^. According to ref. ^41^, different stages of malaria infection decrease the refractive index of a healthy red blood cell that is 1.4. Ring, trophozoite and schizont are three stages of malaria infection. Based on the simulation results, our suggested biosensor has the high sensitivity of 2000 nm/RIU and high FOM of 100 RIU^−1^ for detection of the first stage of malaria infection, which can be so useful for quick treatment of infected people. It is considerable that 250 million people are affected by malaria annually^41^. The bacillus bacteria change the average refractive index of a healthy cell from 1.4 to 1.539. Also, the cancer cells have higher refractive index of 1.370 than the healthy cells (1.353)^41^. The sensing parameters of the proposed biosensor for all mentioned applications are listed in Table 2.

**Table 2:** The sensing parameters of the proposed plasmonic sensor in biomedical applications.

The test material		Refractive index	S (nm/RIU)	FOM (RIU^−1^)
25% solution of glucose in water		*n*_*w*_ = 1.3198, *n*_*g*_ = 1.3594	1457.07	85.71
Malaria infection	Ring stage	*n*_1_ = 1.4, *n*_2_ = 1.395	2000	100
	Trophozoite stage	*n*_1_ = 1.4, *n*_2_ = 1.383	1523.53	67.17
	Schizont stage	*n*_1_ = 1.4, *n*_2_ = 1.373	1344.44	67.22
Bacillus bacteria		*n*_1_ = 1.4, *n*_2_ = 1.539	876.26	43.81
Cancer cells		*n*_1_ = 1.353, *n*_2_ = 1.370	1407.59	77.40

## Analytical Methods

The simulation results of the proposed biosensor can be evaluated by analytical methods. The equivalent circuit model is the first method that is utilized to verify the simulation results. The dielectric substrate is modeled by the transmission line 1 (TL-1) with the characteristic impedance of ${Z}_{s}={\eta }_{0}/\sqrt{{\varepsilon }_{r}}$, the propagation constant of ${\beta }_{s}=2\pi /{\lambda }_{g}$, and the electrical length of ${E}_{s}=2{\beta }_{s}{t}_{s}$, where ${\eta }_{0}$, ${\varepsilon }_{r}$, and ${\lambda }_{g}$ are the free space impedance, the relative permittivity of the dielectric layer, and the guided wavelength, respectively^14^. Also, the test medium, modeled by the transmission line 2 (TL-2), has the same parameters of the TL-1, in which ${\varepsilon }_{r}$ should be replaced by ${\varepsilon }_{a}={n}_{analyte}^{2}$.

**Figure 12:**
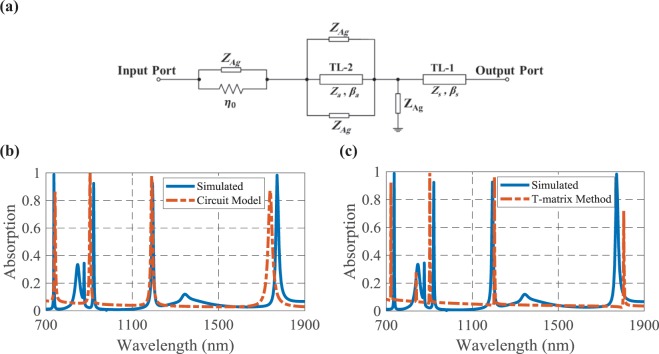
(**a**) The equivalent circuit model of the proposed four-band perfect absorber, the comparison between the simulated and analytical methods of (**b**) equivalent circuit model and (**c**) T-matrix.

The parallel branches that consist of different elements are used to model the MIM and grating configurations, as shown in Fig. 12(a). The Ag impedance can be calculated trough the well-known formula^42^:

2 $${Z}_{Ag}={R}_{Ag}+j\omega {L}_{Ag}=\sqrt{\frac{\omega \mu }{2\sigma }}(1+j)$$

where $\sigma$ and $\mu$ are the conductivity and permeability of the metallic layer, respectively.

The second analytical method is the transmission (*ABCD*) matrix. In this case, the T-matrix of each element of the equivalent circuit is extracted and calculated. The T-lines of the substrate and test medium can be modeled with $${T}_{s}=[\begin{array}{c}\cos ({\beta }_{s}{t}_{s})\\ j\,\sin ({\beta }_{s}{t}_{s})/{Z}_{s}\end{array}\begin{array}{c}j{Z}_{s}\,\sin ({\beta }_{s}{t}_{s})\\ \cos ({\beta }_{s}{t}_{s})\end{array}]$$ and $${T}_{a}=[\begin{array}{c}\cos ({\beta }_{a}{t}_{2})\\ j\,\sin ({\beta }_{a}{t}_{2})/{Z}_{a}\end{array}\begin{array}{c}j{Z}_{a}\,\sin ({\beta }_{a}{t}_{2})\\ \cos ({\beta }_{a}{t}_{2})\end{array}]$$, respectively^43^. The shunt and series branches of the circuit model have the T-matrices of $${T}_{shunt}=[\begin{array}{c}1\\ 1/{Z}_{shunt}\end{array}\begin{array}{c}0\\ 1\end{array}]$$ and $${T}_{series}=[\begin{array}{c}1\\ 0\end{array}\begin{array}{c}{Z}_{series}\\ 1\end{array}]$$, respectively^43^. The transmission matrix of the whole structure can be achieved by cascading the T-matrix of each section. Therefore, the scattering parameters and optical response of the T-matrix model can be obtained by^43^:

3 $$R={|{S}_{11}|}^{2}={|\frac{A+B/{\eta }_{0}-C{\eta }_{0}-D}{A+B/{\eta }_{0}+C{\eta }_{0}+D}|}^{2}$$

where *A*, *B*, *C* and *D* are the elements of the main T-matrix of the proposed structure.

The extracted circuit model is simulated in the Advanced Design System (ADS) software to calculate the scattering parameters. By setting the values of Table 3, the absorption spectrum of the suggested biosensor according to the mentioned analytical methods is calculated through Eq. (1).

**Table 3:** The Parameters values of the analytical methods.

Parameter	Value	Parameter	Value
${Z}_{s}$	$251.32(\varOmega )$	${E}_{s}=2{\beta }_{s}{t}_{s}$	${132.51}^{\circ }$
${Z}_{a}$	$285.64(\varOmega )$	${E}_{a}=2{\beta }_{a}{t}_{2}$	${524.68}^{\circ }$
${R}_{Ag}$	$35(\varOmega )$	${L}_{Ag}$	$0.4({\rm{fH}})$

As shown in Fig. 12(b,c), both analytical methods have good agreement with the simulation results for four resonance wavelengths. Since the dielectric layers of the structure have been modeled by the T-lines, the electrical lengths of the T-lines affect the scattering parameters of the extracted model and the absorption spectra of Fig. 12. The results of the analytical methods can be tuned through the optimization and changing the values of the extracted parameters of Table 3 around their initial values. Therefore, the absorption spectra of the analytical methods can be changed to have more resonances and match with all resonance wavelengths of the simulation results. But, the absorption value may be diminished. Therefore, there is a trade-off between the resonance wavelengths and their absorption values. Here, we have focused on the four resonance wavelengths that their sensing performance have been considered and so in the extracted circuit model we have tried to match these four resonance wavelengths well.

## The Switching Capability of the Proposed Biosensor

**Figure 13:**
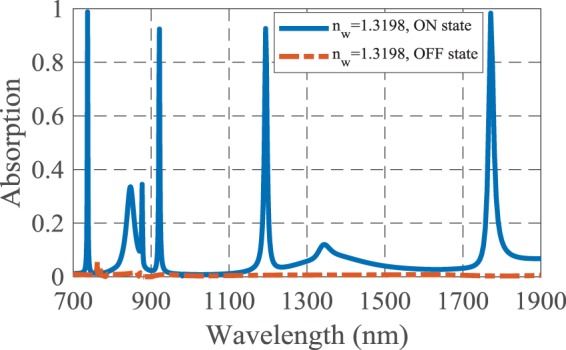
The absorption spectra of the proposed plasmonic biosensor for “ON” and “OFF” states when the electric field polarization changes from y- to x-axis, respectively.

According to the design procedure, the presented structure can be illuminated by an incident lightwave with y-polarized E-field, which is perpendicular to the Ag-air grating. Consequently, the change of incident wave polarization from y- to x-axis changes the performance of the absorber and leads to total reflection of the incident wave. Therefore, the absorption coefficient becomes near-zero for the whole wavelength range of 700 to 1900 nm, as depicted in Fig. 13. This figure shows the switching capability of the proposed biosensor from “ON” to “OFF” state.

The response time of the suggested plasmonic switch can be calculated by applying a continuous wave (CW) at the resonance wavelength to the structure and time monitoring of the output port, when the output signal approaches the steady state. The process of changing the incident lightwave polarization does not be considered in calculation of the response time. Another main parameter of a plasmonic switch is extinction ratio that exhibits the difference between the absorption values in “ON” and “OFF” states^37^:

4 $$\eta ({\rm{dB}})=10\,\log \left(\frac{{A}_{on}}{{A}_{off}}\right)$$

To have a better insight of simultaneous sensing and switching performances of the presented perfect absorber, the parameters of both capabilities for detection of glucose in water are listed in Table 4.

**Table 4:** The sensing and switching performances of the proposed biosensor for detection of glucose in water.

*λ* (nm)	S (nm/RIU)	FOM (RIU^−1^)	*η* (dB)	Response time (fs)
745	479.79	239.89	21.50	180
935	686.86	171.71	21.20	170
1207.5	878.78	109.84	21.88	150
1800	1457.07	85.71	25.15	100

Our suggested biosensor has the capabilities of sensing and switching, simultaneously. To the best of our knowledge, there is not any reported plasmonic structure with two mentioned abilities in one designed structure. Therefore, our proposed structure is compared with the plasmonic sensors and switches in near-infrared region, separately. Table 5 shows the comparison between the performances of our suggested structure with previous works. Some reported structures may have high sensitivity or FOM in sensing performance and high extinction ratio or fast response time in switching performance, but not simultaneously. In contrast, our suggested structure not only has two capabilities, simultaneously, but also has high sensing and switching performances.

**Table 5:** Comparison of the sensing and switching performances of our proposed biosensor with other reported plasmonic sensors and switches.

Reference	S (nm/RIU)	FOM (RIU^−1^)	*λ* (nm)	*η* (dB)	Response Time (fs)
^28^	1890.4	—	1535.7	—	—
^30^	1061	—	1554	—	—
^31^	893.5	—	1550	—	—
^36^	1562.5	38.6	1550	—	—
^44^	557	6.1	1000	—	—
^45^	503	63	930	—	—
^46^	223	19.5	1170	—	—
^47^	250	28	1100	—	—
^48^	600	28	1100	—	—
^49^	1100	224	1000	—	—
^50^	1200	15	1550	—	—
^37^	—	—	1550	9.27	—
^51^	—	—	1550	13.96	90
^52^	—	—	1550	11.14	—
^53^	—	—	1550	14.36	—
^54^	—	—	1550	20	14e9
This work	2000	100	1880	22.13	200
	1457.07	85.71	1800	25.15	100

## Conclusion

In summary, we have investigated a plasmonic biosensor with switching capability in near-infrared region. The suggested structure has the high sensitivity of 2000 nm/RIU and high FOM of 100 RIU^−1^ due to the MIM coupling and interaction between the Ag cubic resonators and test medium. The simulation results show the ability of the proposed plasmonic sensor in detection of glucose in water, diagnosis of malaria infection, bacillus bacteria and cancer cells. The Ag-air grating, which has been used to excite the SPs in the plasmonic sensor, helps the structure to have switching capability with high extinction ratio of 25.15 dB and fast response time of 100 fs. Therefore, the implemented structure has better performance than the other reported plasmonic sensors and switches in two considered capabilities of sensing and switching, simultaneously. The simulation results have been validated by two analytical methods. The equivalent circuit model and T-matrix methods are two utilized analytical methods, which have been used in near-infrared region in this work. The multi-application idea of this manuscript, considering the fabrication feasibility and verifying the simulated results with analytical methods can be used for plasmonic devices in the future.
